# Comorbidity Study of Attention-deficit Hyperactivity Disorder (ADHD) in Children: Applying Association Rule Mining (ARM) to Korean National Health Insurance Data

**Published:** 2018-04

**Authors:** Leejin KIM, Sungmin MYOUNG

**Affiliations:** 1. Dept. of Child Studies, Chonbuk National University, 567 Baekje-daero, Deokjin-gu, Jeonju-si, Jeollabuk-do, South Korea; 2. Dept. of Health Administration, Jungwon University, 85 Munmu-ro Goesan-eup Goesan-gun, Chungbuk, South Korea

**Keywords:** ADHD, Association rule mining, Comorbidity, Data mining

## Abstract

**Background:**

The aim of this study was to explore the comorbidity of Attention-Deficit Hyperactivity Disorder (ADHD) for the Korean national health insurance data (NHID) by using association rule mining (ARM).

**Methods:**

We used data categorized mental disorder according to the international classification of disease, 10^th^ revision (ICD-10) diagnosis system from NHID from 2011 to 2013 in youths aged 18 yr or younger. Overall, 211420 subjects, comorbid cases with ADHD were present in 105784. ARM was applied to the Apriori algorithm to examine the strengths of associations among those diagnosed, and logistic regression was used to evaluate the relations among rules.

**Results:**

The most prevalent comorbid psychiatric disorder of ADHD youths was mood/affective disorders. From results of ARM, nine association rules (support≥1%, confidnce≥50%) were produced. The highest association was found between specific developmental disorders of scholastic skills and ADHD. Among association of three comorbid diseases, tic disorder was an important role in the association between ADHD and other comorbid diseases through results of ARM and logistic regression.

**Conclusion:**

The practical application of ARM for discovering the comorbidity of ADHD in large amount real-data such as the Korean NHID was mostly confirmed by past studies. The results of this study will be helpful to researchers evaluating the stability of their diagnosis in ADHD.

## Introduction

Attention-Deficit Hyperactivity Disorder (ADHD) is the most common mental disorders that develop in children. The presence of ADHD in children has been known to give negative effects for many aspects of life, such as self-esteem, academic performance, social functions, and parent-child relationships (1).

Many studies have suggested estimating the prevalence of ADHD. In the United States, the prevalence of ADHD is generally estimated 3%–10% (2).

According to American Psychiatric Association, the prevalence of ADHD in school-aged children ranges 3%–7%. Furthermore, residual or full symptoms of ADHD is continued to adulthood in 30%–60% (3, 4). In India, researchers reported an estimated prevalence of ADHD 10.3%–13.6% for 4–16 ages, and community studies in Yeman suggested that an estimated prevalence of range 11.7%–20.2% for 7∼10 yr old school children (5–7). In Korea, school-based mental health services represented 6.5% prevalence for epidemiologic research. Moreover, the estimated prevalence based the DSM-IV disorders for 1645 children were 4.8%–7.0% (8).

A substantial proportion of patients with ADHD also have been reported comorbidity with psychiatry disorders other than ADHD, such as anxiety disorder, mild mental retardation, depression neurosis, major depressive disorder, autism and bipolar disorder (9–11).

Inspecting the associations of comorbidities based on diagnostic data will be useful in predicting their risk and thus more effectively treating patients with ADHD. Many comorbidity studies of ADHD related to association of dual-diagnosis or estimation of the prevalence of co-occurring of two diagnoses and three or more comorbidities have been applied some data mining techniques (12).

We consider the relationship among comorbidities of ADHD based on association rule mining (ARM) among these data mining techniques.

ARM, also known as market basket analysis, is the discovery of association relationships or correlations among a set of attributes (items) in a database (13). It applied to pattern mining fields, such as lifestyle risk behavior patterns, clustering to identify related questionnaire data, gene expression data, and cancer prevention factors (14–17). In the field of epidemiology, the important thing of ARM was proposed that the value of ‘confidence’ is arithmetically equal to the ‘comorbidity’ (12).

The Korean National Health Insurance (KNHI) established in 2000, is a single-payer program and currently has served mandatory social insurance system for all residents in Korea. The National Health Insurance Data (NHID) is provided by KNHI represents the entire Korean population and can be used as a population-based database. The Korean NHID consists of four databases of the insured that contain data on health checkups, health insurance claims and long-term care insurance (LTCI) (18).

Researchers can be analyzed the Korean NHID for policy and academic research purposes after the Institutional Review Board approval. Identifying the incidence or prevalence of specific disease by using Korean NHID have been studied and published in some previous study (19,20), but none of them focused on comorbidity of ADHD. This study aimed to explore the comorbidity of ADHD, and determine relations among two or more comorbidities by using the technique of ARM. It is expected that not only to show the usefulness of ARM in large amount of medical database such as the Korean NHID but also to get valuable insight about the network associations among ADHD comorbidity in Korean population.

## Methods

### Study Population and Data Collection

**Fig. 1:**
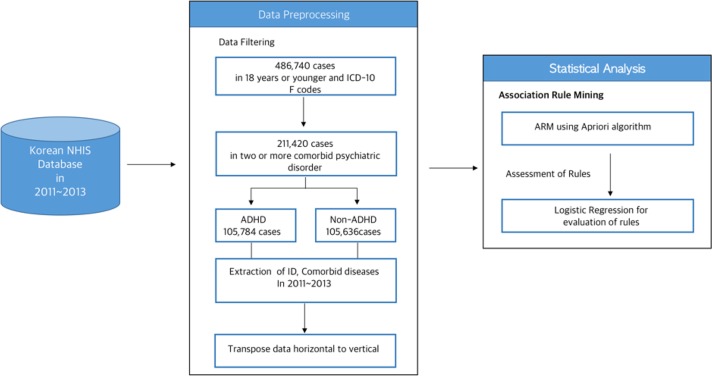
Schematic diagram of the study workflow

This study utilized the Korean NHID from 2011 to 2013. This dataset shows information including socioeconomic characteristics, health examinations, medical care institution data, and details of medical treatment and disease classification codes based on the International Classification of Diseases, 10th revision (ICD-10) (Fig. 1). We examined subjects who identified 486740 youths aged 18 or younger and ICD-10 ‘F’ code (mental and behavioral disorders) from 2011 to 2013. Among cases with psychiatric disorders, we identified mood/affective disorders (ICD-10 codes: F30.x, F31.x, F32.x, F33.x, F34. x, F38.x and F39.x), anxiety disorders (F40.x, F41.x, F42.x, F43.x), mild/moderate mental retardation (F70.x and F71.x), specific developmental disorders of speech and language (F80.x), specific developmental disorders of scholastic skills (F81.x), pervasive developmental disorders (F84.x), conduct disorders (F91.x), mixed disorders of conduct and emotions (F92.x), emotional disorders with onset specific to childhood (F93.x), tic disorders (F95.x) and other behavior and emotional disorders with onset usually occurring (F98.x). From among the data, 211420 subjects with two or more comorbid psychiatric disorders were selected and consisted of 105784 (50.04%) ADHD and 105636 (49.96%) non-ADHD.

The present study was approved by the Institutional Review Board of the Chonbuk National University (IRB No: 2016-01-007) in Jeonbuk, South Korea.

### Analysis Method

The proposed framework is to focus on the investigating the associations of the comorbid diseases with ADHD. We considered ARM, also known as market basket analysis that is a popular data mining method.

A transaction (T) is denoted a collection of ADHD /non-ADHD comorbid records in database of Korean NHID. In this transaction, D1 and D2 denote diseases that enrollees have. Formally, an association rule generated from denoted ‘D1 → D2’, it means the appearance of D1 implies the appearance of D2 in the same transaction. A rule does not have to imply cause and effect necessarily. The measures for ARM are the values of support, confidence, and lift of the rule. The support for the rule (D1 → D2) is defined as the probability that contains both D1 and D2.

[1] $$\text{support}(\%)=\frac{\text{Number}\;\text{of}\;\text{disease}\;\text{D}1\cap \text{D}2}{\text{Total}\;\text{number}\;\text{of}\;\text{disease}}$$

The confidence for D1 → D2 is the conditional probability of D2 given that a person has diagnosis D1.

[2] $$\text{confidence}(\%)=\frac{\text{Number}\;\text{of}\;\text{disease}\;\text{D}1\cap \text{D}2}{\text{Number}\;\text{of}\;\text{disease}\;\text{D}1}$$

The lift of the rule D1 → D2 is the ratio of the confidence of the rule to the expected confidence, assuming the diagnoses are independent. The lift is interpreted as a measure of importance of a rule. The lift value greater than 1 indicates a positive effect; smaller than 1 indicates a negative effect, near 1 indicates no effect.

[3] $$\text{Lift}(\text{D}1\to \text{D}2)=\frac{\text{Confidence}(\text{D}1\to \text{D}2)}{\text{Support}(\text{D}2)}=\text{Confidence}(\text{D}1\to \text{D}2)\times \frac{\text{Total}\;\text{number}\;\text{of}\;\text{disease}}{\text{number}\;\text{of}\;\text{D}2}$$

**Fig. 2:**
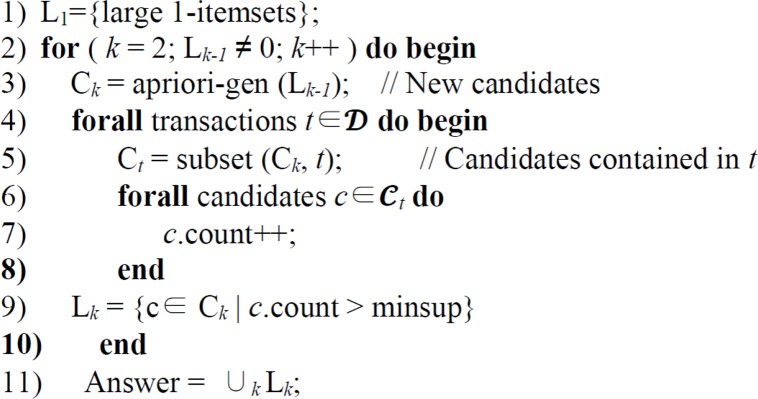
Apriori algorithm

Apriori algorithm, the most commonly used ARM rule, computes the frequent item sets in the database through several iterations. It was introduced by Agrawal and Srikant (21), and classical Apriori algorithm is provided in Fig. 2.

The ARM is fundamentally based on two-step principles (21–23). In the first step, find all the frequent item sets, which have more than minimum support in the transaction database. In second step process, generate strong association (high confidence) rules from frequent item sets.

The first pass of the algorithm is to calculate the support for the large 1-itemsets, L_*1*_, as in line 1. From this point, the algorithm applies k number of iterations (line 2 in Fig. 2).

Each subsequent pass uses the item sets discovered in the previous pass whose items have supported greater than the user-defined minimum support. This set of item sets is used to generate the candidate item sets C_*k*_, using the apriori candidate generation (apriori-gen) algorithm. The idea of the apriori-gen algorithm is to generate all the supersets of the large *k*-items sets from all the (*k*-1)-item sets (line 3 in Fig. 2). It works by generating the candidate item set by two-step procedure (join and prune steps). The join step joins two frequent (*k*-1)-item sets to generate a candidate c. The two frequent L_*k*-1_ item sets have exactly same items except the last one. After the join step, the candidate item sets C_*k*_ are generated. The pseudo SQL code for this step is given below (21):

**insert** into C_*k*_
**select** *p*.item_1_, *p*.item_2_, …, *p*.item_k-1_, *q*.item_*k-1*_
**from** L_*k-1*_ *p*, L_*k-1*_ *q*
where *p*.item_1_ = *q*.item_1_, …, *p*.item_k-2_= *q*.item_*k-2*_, *p*.item_*k-1*_<*q*.item_*k-1*_;

The second step is the prune steps, which remove all item, sets *c* ∈ C_*k*_ such that some (*k*-1)-subest of *c* is not in L_*k*-1_. This step can be seen below:

**forall** itemsets *c* ∈ C_*k*_ **do**
**forall** (*k*-1)-subsets *s* of *c* **do**
**if** (*s* ∉ L_*k-1*_) **then**
**delete** *c* from C_*k*_

After generating candidate item sets, the transaction database is scanned and the support of candidates in C_*k*_ is calculated (lines 4–8 in Fig. 2). At the end of the scan, it determines actually frequent item sets among candidates.

After determining the association rules, we applied a multiple logistic analysis to evaluate the relations among rules. Odds ratio (OR) and corresponding 95% confidence intervals (CI) were indicated.

Data management and analysis were used SAS v9.3 and R-package 3.4.0. Statistical significance was considered for *P*-values under 0.05.

## Results

The most prevalent comorbid psychiatric disorder (Table 1) of ADHD youths (18 yr or younger) was mood/affective disorders group 32.34%, followed by anxiety disorders group 20.98%, emotional disorders with onset specific to childhood 17.76%, tic disorders 16.17%, conduct disorders 10.37%, mild mental retardation 7.91%, pervasive developmental disorders 6.37%, specific developmental disorders of speech and language 5.40%, mixed disorders of conduct and emotions 4.68% and specific developmental disorders of scholastic skills 3.05%.

**Table 1:** High frequency^*^ comorbid diseases with ADHD^**^ (N=105,784)

***Comorbid Psychiatry Disease***	***ICD-10 Code***	***Comorbidity (%)***
Mood(Affective) Disorders	F30.x∼F34.x, F38.x∼F39.x	32.34
Anxiety Disorders	F40.x∼F43.x	20.98
Emotional Disorders with onset specific to childhood	F93.x	17.76
Tic Disorders	F95.x	16.17
Conduct Disorders	F91.x	10.37
Mild Mental Retardation	F70.x	7.91
Pervasive Developmental Disorders	F84.x	6.37
Other Behavioral and Emotional disorders	F98.x	6.31
Mixed Disorders of Conduct and Emotions	F92.x	4.68
Specific Developmental Disorders of Scholastic Skills	F81.x	3.05

* is defined more than 3% //

** ADHD: Attention Deficit Hyperactivity Disorder

The results of ARM between ADHD and specific psychiatric disorders listed in Table 2. For learning association rules, we established a support threshold of 1% and confidence thresholds of 50%. There are 9 association rules satisfied these thresholds.

**Table 2:** Results of ARM of psychiatric disorders comorbid among ADHD children (18 yr or younger)

Rule^*^	***Lift***	***Support (%)***	***Confidence (%)***
Specific DevDis → ADHD	1.45	1.53	72.41
Conduct Disorders → ADHD	1.33	5.19	66.55
Tic Disorders → ADHD	1.33	8.09	66.34
EmotDis → ADHD	1.25	8.88	62.71
MixedDis → ADHD	1.09	2.34	54.76
Tic Disorders & MoodDis → ADHD	1.07	1.59	53.48
Tic Disorder & AnxDis → ADHD	1.05	1.36	52.61
Other BeEmotDis → ADHD	1.05	3.16	52.28
Conduct Disorders & MoodDis → ADHD	1.03	1.57	51.53

* Rules were sorted by lift values in descendant order

Note: Specific DevDis: Specific Developmental Disorders of Scholastic Skills; EmotDis: Emotional disorders with onset specific to childhood; MixedDis: Mixed Disorders of Conduct and Emotions; MoodDis: Mood/Affective Disorders; AnxDis: Anxiety Disorders; Other BeEmotDis: Other Behavioral and Emotional Disorders with onset

The highest association was the rule ‘specific developmental disorders of scholastic skills → ADHD’ with confidence 72.41% which means ratio of the co-occurrence rate of specific developmental disorders of scholastic skills and ADHD over the prevalence of specific developmental disorders of scholastic skills. The support value of 1.53% indicates that prevalence rate of both specific developmental disorders of scholastic skills and ADHD within a certain period. The lift value of 1.45 means specific developmental disorders of scholastic skills were positively associated with ADHD. The second was ruled ‘conduct disorders → ADHD’ with confidence 66.55% and support 5.19%. The other rules were ‘tic disorders → ADHD’ (confidence: 66.34%, support 8.09%), ‘emotional disorders with onset specific to childhood → ADHD’ (confidence: 62.71%, support 8.88%), ‘mixed disorders of conduct and emotions → ADHD’ (confidence: 54.76%, support: 2.34%).

All lifts of rules were interpreted positively associated with ADHD.

Among association of three comorbid diseases, tic disorder was an important role in association between ADHD and other comorbid diseases, such as mood/affective disorders and anxiety disorders.

**Fig. 3:**
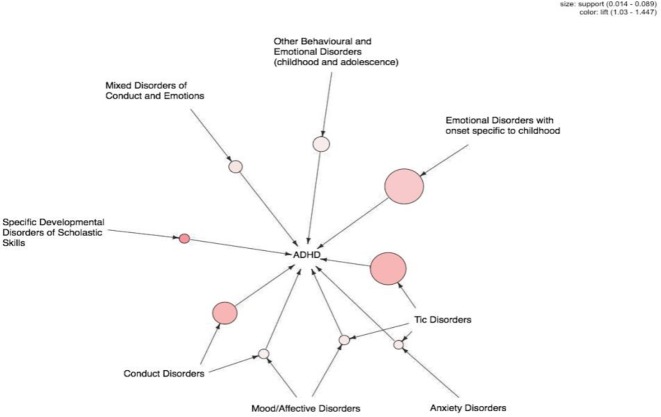
Visualization of 9 association rules

In Fig. 3, we have illustrated a visual representation of 9 association rules. In coming to edges to the grey colored node, show the left-hand side (comorbid disease) of the association rule while outgoing edge represents right-hand side (ADHD). The color of edge means to lift, which indicated importance of association, and the size of edge indicates the support of association. For our analysis, Emotional disorders with onset specific to childhood, tic disorder, and conduct disorders were the largest, and the lines linking tic disorder with both mood/affective disorders and 1 anxiety disorder were most noticeable.

Considering the left side of the association rules as explanatory variable, and the right side of the rules as response variables, we present adjusted odds ratio for sex and age with their 95% confidence intervals from the logistic regression to evaluate the ARM results (Table 3). All of association rules had an odds ratio (OR) greater than 1 and the highest OR was the specific developmental disorders of scholastic skills (OR=2.19, 95% CI=[2.04, 2.34]), followed by conduct disorder (OR=2.13, 95% CI=[2.05, 2.20]) and tic disorder (OR=1.59, 95% CI=[1.54,1.63]). After controlling for mood/affective disorder, sex, and age, conduct disorder was significantly associated (OR=2.09, 95% CI=[2.02, 2.16]).

**Table 3:** Results of logistic regression analysis for ADHD

***Predictors***	OR^*^ (95% CI)
Specific DevDis	2.19 (2.04–2.34)
Conduct Disorders	2.13 (2.05–2.20)
Conduct Disorders and MoodDis	2.09 (2.02–2.16)
Tic Disorders	1.59 (1.54–1.63)
Tic Disorder and AnxDis	1.54 (1.50–1.59)
Tic Disorders and MoodDis	1.51 (1.46–1.55)
EmotDis	1.42 (1.39–1.46)
MixedDis	1.35 (1.29–1.41)
Other BeEmotDis	0.85 (0.82–0.88)

* OR: Odds Ratio was adjusted for sex and age

Tic disorder with anxiety disorder was observed higher odds ratio (OR=1.54) than with mood/affective disorder (OR=1.51). In addition, OR for tic disorder with controlling adjusted comorbid disease (anxiety disorder or mood/affective disorders) appeared similar to unadjusted comorbid disease.

## Discussion

ADHD have known as high risk of comorbidity with other illness, especially the psychiatric diseases (12). This study was the first research to investigate the association between ADHD and psychiatric comorbid diseases in Korean NHID. The most frequently occurring comorbid disease among ADHD was mood/affective disorders group. It is quite capable of other researches suggestion with 25%–48% of ADHD patients had comorbid conditions (20).

From the ARM results, mood/affective disorders show to be associated with ADHD and other prevalent comorbid diseases, such as tic disorder and conduct disorders. In addition, associated comorbid diseases, including tic disorder and anxiety disorders can be contributors to ADHD. These results reveal to be compatible with the previous studies for comorbidities of ADHD (24). In the Taiwanese study using NHID, developmental delay (DD) was discovered as an important role between ADHD and other psychiatric disorders, like anxiety disorders, depression, and autism (12). Although DD was not presented different from Taiwanese study, other psychiatric disorders, such as anxiety disorder and mood/affective disorders including depression were associated with ADHD in Korean NHID study.

In this study, the logistic regression analysis was presented that the risk pattern distribution of the ADHD group and non-ADHD group were given by OR. The three most frequently OR for comorbid disease among ADHD were specific developmental disorders of scholastic skills, conduct disorder, and tic disorder. These results seem to be relatively lower than other researches because the study population is limited to more than two comorbid psychiatric diseases.

There are several limitations on this investigation. First, the comorbidity rate of each psychiatric disorder in this study is lower than in previous studies. It seems to be limitations of our data source as clinical referral patterns. Therefore, our future research will be investigating more large data, such as long-term (more 3 yr) Korean NHID or Health Insurance Review and Assessment (HIRA) database. Second, this study is not able to consider causal relationship because of cross-sectional data.

## Conclusion

The highest association between ADHD and comorbid diseases was tic disorders, which is compatible with previous neurologic and epidemic studies. Our study also demonstrated the usefulness of ARM in ADHD comorbidity study in a large-scale, nationally representative dataset, namely the Korean NHID. The result of this study will be helpful to researchers evaluating the stability of their diagnosis with ADHD, and still, need some further evidence for establishing causal relationships.

Future research focuses on experiment with summarization of ARM results into a predictive model such as Bayesian network or decision tree model.

## Ethical considerations

Ethical issues (Including plagiarism, informed consent, misconduct, data fabrication and/or falsification, double publication and/or submission, redundancy, etc.) have been completely observed by the authors.
